# Efficient Optosensing of Hippuric Acid in the Undiluted Human Urine with Hydrophilic “Turn-On”-Type Fluorescent Hollow Molecularly Imprinted Polymer Microparticles

**DOI:** 10.3390/molecules28031077

**Published:** 2023-01-20

**Authors:** Wanlan Zhang, Qun Li, Huiqi Zhang

**Affiliations:** State Key Laboratory of Medicinal Chemical Biology, Key Laboratory of Functional Polymer Materials (Ministry of Education), Collaborative Innovation Center of Chemical Science and Engineering (Tianjin), Frontiers Science Center for New Organic Matter, and College of Chemistry, Nankai University, Tianjin 300071, China

**Keywords:** molecularly imprinted polymers, hollow, complex biological samples, fluorescence “turn-on”, hippuric acid, human urine sample, surface-initiated ATRP, sacrificial template method

## Abstract

The development of complex biological sample-compatible fluorescent molecularly imprinted polymers (MIPs) with improved performances is highly important for their real-world bioanalytical and biomedical applications. Herein, we report on the first hydrophilic “turn-on”-type fluorescent hollow MIP microparticles capable of directly, highly selectively, and rapidly optosensing hippuric acid (HA) in the undiluted human urine samples. These fluorescent hollow MIP microparticles were readily obtained through first the synthesis of core-shell-corona-structured nitrobenzoxadiazole (NBD)-labeled hydrophilic fluorescent MIP microspheres by performing one-pot surface-initiated atom transfer radical polymerization on the preformed “living” silica particles and subsequent removal of their silica core via hydrofluoric acid etching. They showed “turn-on” fluorescence and high optosensing selectivity and sensitivity toward HA in the artificial urine (the limit of detection = 0.097 μM) as well as outstanding photostability and reusability. Particularly, they exhibited much more stable aqueous dispersion ability, significantly faster optosensing kinetics, and higher optosensing sensitivity than their solid counterparts. They were also directly used for quantifying HA in the undiluted human urine with good recoveries (96.0%–102.0%) and high accuracy (RSD ≤ 4.0%), even in the presence of several analogues of HA. Such fluorescent hollow MIP microparticles hold much promise for rapid and accurate HA detection in the clinical diagnostic field.

## 1. Introduction

Molecularly imprinted polymers (MIPs) are synthetic receptors with nanosized target analyte-binding cavities [1,2,3,4,5,6]. They can be readily prepared via the simple template-directed synthetic strategy. Their outstanding attributes (i.e., high molecular recognition ability, excellent physiochemical stability, facile preparation, low cost, and easy functionalization) make them highly promising substitutes for biological receptors (e.g., antibody and enzyme) in the sensor area [7,8,9,10,11,12]. In particular, MIP-based fluorescent chemosensors combining the advantages of MIPs and fluorescent analyses (i.e., high sensitivity, simple instruments, and easy implementation [13]) have attracted enormous interest in the bioanalytical and biomedical fields [7,8,9,10,11,12]. They are normally fabricated by simply incorporating various fluorescent species into MIPs, where the fluorescent species function as the transducers to quantitatively transform the recognition processes of the MIPs into detectable photosignals. Despite the tremendous progress made in the development of MIP-based optosensors, the fluorescent MIPs that can be directly used for selective optosensing of small organic analytes in the complex biological samples are still rare, which greatly limits their broad, real-world applications.

To address the above-mentioned challenging issue, our group has developed some versatile strategies for preparing complex biological sample-compatible fluorescent MIPs through grafting hydrophilic polymer brushes onto the surfaces of the fluorescent MIP micro/nanoparticles (labeled with either an organic fluorescent unit or inorganic CdTe quantum dots (QDs) [14,15,16] or both of them [17,18,19]) via various controlled/“living” radical polymerization techniques. The resulting hydrophilic single fluorescent MIPs could directly and selectively detect antibiotic tetracycline (Tc) [14,16] and food additive folic acid (FA) [15] in the complex biological samples (including the undiluted pure serums [14,15] or both the undiluted pure serums and milks [16]) through fluorescence quenching (or “turn-off”) mechanism, while the obtained hydrophilic dual (or ratiometric) fluorescent MIPs were capable of directly and selectively optosensing (even with the naked eyes) the herbicide 2,4-dichlorophenoxyacetic acid (2,4-D) in the undiluted pure milks through the fluorescence “turn-on” mechanism [17,18,19]. Note that the “turn-on”-type fluorescence of the optosensors can avoid false-positive responses owing to less interfering effects and achieve higher sensitivity due to their lower optical background and higher signal-to-noise ratio in comparison with fluorescence quenching [20]. In all these cases, the hydrophilic polymer brushes function as a protective layer for the fluorescent MIP particles, which can not only significantly reduce the hydrophobicity-induced nonspecific bindings of the fluorescent MIPs in aqueous media by enhancing their surface hydrophilicty, but they also improve their antifouling ability and thus prevent the proteins in the complex biological samples to accumulate on their surfaces and block the imprinted binding sites [21,22,23]. Nevertheless, the presence of hydrophilic polymer brushes on the MIP particle surfaces has proven to retard their analyte binding kinetics, mainly because of the barrier effect of the polymer brush layers on the diffusion of the target analytes to the imprinted binding sites on the MIP particles [24]. Such retarded analyte binding kinetics will largely reduce the optosensing speed of the fluorescent MIPs and thus has negative influence on their practical uses. Therefore, the development of complex biological sample-compatible fluorescent MIPs with more rapid optosensing kinetics is highly desirable. In addition, the dispersion stability of such complex biological sample-compatible fluorescent MIP particles in the complex aqueous solutions still needs to be improved to facilitate the handling of the samples and provide more repeatable detection results.

Thus far, some useful approaches have been developed to enhance the binding kinetics of the MIPs. One normally used approach is to prepare core-shell-structured MIP particles by grafting a thin MIP layer onto various solid particle surfaces via different synthetic strategies [25,26,27]. The resulting MIPs have easily accessible imprinted binding sites that are in close proximity to the MIP particle surfaces, which can thus largely enhance the template binding kinetics. Another efficient approach has also been developed to further improve the binding kinetics of such core-shell-structured MIP particles by etching their cores [28,29,30,31]. The resulting hollow MIP particles were found to require much less time to reach the equilibrium binding in comparison with their solid counterparts. In particular, these hollow MIP particles are also expected to have improved dispersion stability in the solutions because of their low densities, which is highly beneficial to their optosensing applications. Nevertheless, to the best of our knowledge, no hollow MIP particles capable of directly and highly selectively recognizing small organic analytes in the complex biological samples have been reported up to now.

Herein, we report on, for the first time, the development of complex biological sample-compatible “turn-on”-type fluorescent hollow MIP microparticles with enhanced optosensing performances by combining our recently developed one-pot surface-initiated atom transfer radical polymerization (ATRP) (SI-ATRP) strategy [19] and sacrificial template method. Hippuric acid (HA) was chosen here as the model target analyte because it is a major human metabolite in toluene-exposed humans (thus as an important biological indicator for occupational exposure monitoring) [32] and has also been recognized as a lung cancer biomarker in human plasma and urine samples [33]. The successful synthesis of such fluorescent hollow MIP microparticles was verified by the characterization results of their morphologies, chemical structures, surface hydrophilicity, and aqueous dispersion stability. Both their presence of imprinted binding sites and complex aqueous sample-compatibility were confirmed by the equilibrium/competitive binding and fluorescent optosensing results in the artificial urine. In particular, their direct, highly selective, rapid, and accurate quantification of HA in the undiluted human urine samples (even in the presence of several analogues of HA) was also demonstrated. To our knowledge, this is not only the first report on the successful preparation of complex biological sample-compatible fluorescent hollow MIP particles but also the first MIP capable of directly and highly selectively detecting HA in the undiluted complex biological samples.

## 2. Results and Discussion

### 2.1. Synthesis and Characterization of the Hydrophilic Fluorescent Solid and Hollow HA-MIP/CP Microparticles

**Scheme 1:**
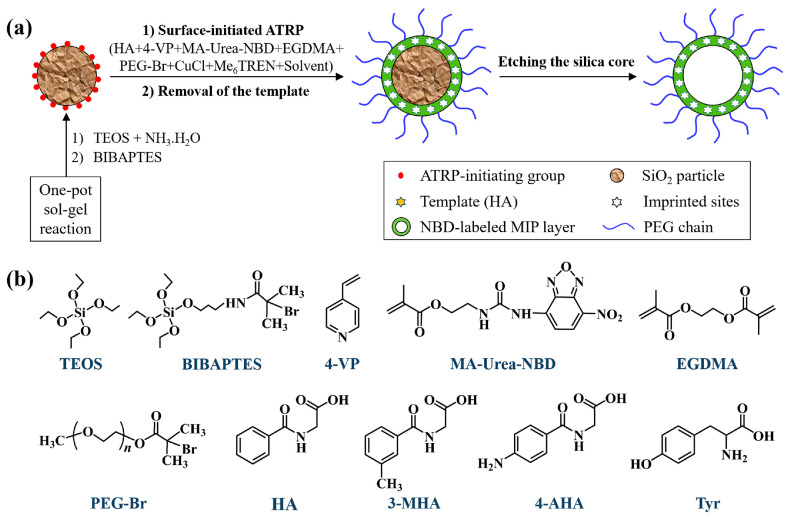
(**a**) Schematic illustration for preparing “turn-on”-type fluorescent hollow MIP microparticles with PEG brushes via the combined use of one-pot SI-ATRP strategy and the sacrificial template method. (**b**) Chemical structures of some reagents utilized in this work.

The aim of this work is to develop complex biological sample-compatible fluorescent MIP microparticles with enhanced optosensing performances. To realize this goal, hydrophilic fluorescent hollow MIP microparticles (i.e., H@NBD-MIP@PEG, entry 4 in Table 1) were prepared through first one-pot synthesis of core-shell-corona-structured hydrophilic fluorescent solid MIP microspheres (i.e., SiO_2_@NBD-MIP@PEG, entry 2 in Table 1) and subsequent removal of their silica core via HF etching (Scheme 1). SiO_2_@NBD-MIP@PEG microspheres were readily obtained via the controlled grafting of a NBD-labeled ultrathin HA-MIP layer with hydrophilic polymer brushes onto the preformed uniform “living” silica particles with surface-bound alkyl halide groups (i.e., ATRP-initiating groups) (prepared via one-pot sol-gel reaction of TEOS in the presence of BIBAPTES (Scheme 1b) [34]) via one-pot SI-ATRP in the presence of PEG-Br (Scheme 1b), where HA, 4-VP, MA-Urea-NBD, and EGDMA were utilized as the template, functional monomer, fluorescent comonomer, and cross-linker, respectively (Scheme 1b), according to our previous reports [17,18,19]. The carboxylic acid and amide groups of HA can form hydrogen bonding interactions with both the pyridine unit of 4-VP and ureido unit of MA-Urea-NBD, which can result in the formation of the self-assemblied HA/4-VP and HA/MA-Urea-NBD supramolecular complexes during the molecular imprinting process. In particular, MA-Urea-NBD could show “turn-on”-type fluorescence upon exposure to HA (Figure S1), which is important for obtaining fluorescent MIP optosensors that can avoid false-positive responses and achieve higher sensitivity [20]. The hydrophilic fluorescent solid control polymer particles (i.e., SiO_2_@NBD-CP@PEG, entry 3 in Table 1) were also similarly prepared, except for omitting HA during the SI-ATRP process. The resulting hydrophilic fluorescent solid HA-MIP and CP showed certain weight increases compared with the starting SiO_2_-Br (entries 1–3, Table 1), revealing that the above one-pot SI-ATRP processes indeed took place.

Hydrophilic fluorescent hollow HA-MIP/CP microparticles (i.e., H@NBD-MIP/CP@PEG, entries 4 and 5 in Table 1) were then directly prepared by etching the silica core from SiO_2_@NBD-MIP/CP@PEG particles (entries 2 and 3, Table 1) with a 10% HF solution in ethanol at ambient temperature [34]. The resulting H@NBD-MIP/CP@PEG exhibited large weight decrease in comparison with their starting solid counterparts, indicating the successful removal of the silica core from SiO_2_@NBD-MIP/CP@PEG.

**Figure 1:**
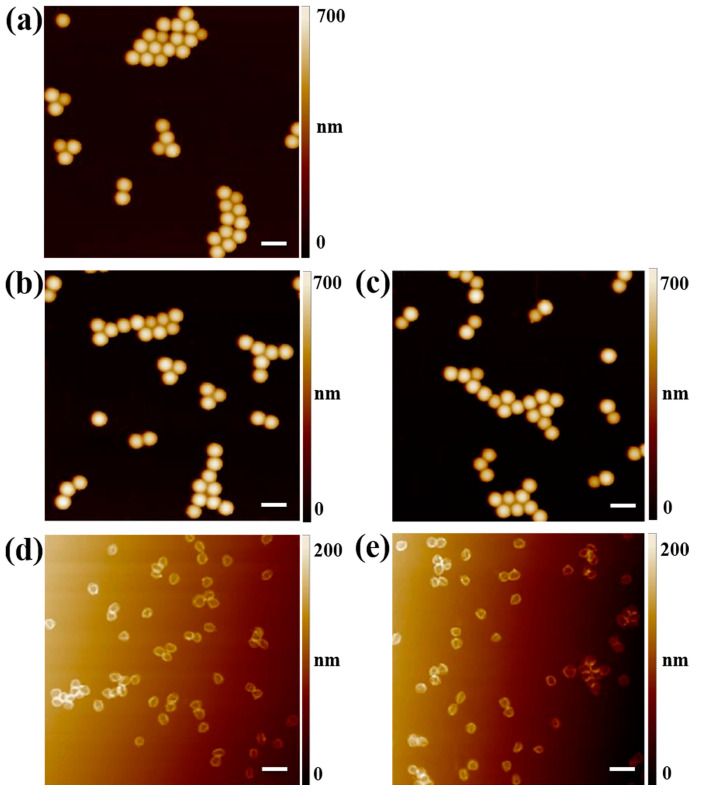
(**a**) AFM height images of SiO_2_-Br (**a**), SiO_2_@NBD-MIP@PEG (**b**), SiO_2_@NBD-CP@PEG (**c**), H@NBD-MIP@PEG (**d**), and H@NBD-CP@PEG (**e**). The scale bar is 1 μm.

AFM characterization revealed that SiO_2_-Br and SiO_2_@NBD-MIP/CP@PEG were all narrowly dispersed spherical microparticles (Figure 1a–c). The diameters of SiO_2_@NBD-MIP/CP@PEG determined by AFM (*D*_n,AFM_) proved to be larger than that of SiO_2_-Br (Table 1), suggesting the successful one-pot SI-ATRP processes. H@NBD-MIP/CP@PEG were found to be uniform collapsed bowl-shaped microparticles with shrinked sizes (compared with their solid counterparts) in their dry state (Figure 1d,e), which again confirmed the successful removal of the silica core. DLS measurements also confirmed the successful synthesis of SiO_2_@NBD-MIP/CP@PEG with their hydrodynamic diameters (*D*_n,DLS_) larger than that of SiO_2_-Br (note that *D*_n,DLS_ values are somewhat larger than *D*_n,AFM_ values (Table 1), as reported previously [19,21]). In addition, H@NBD-MIP/CP@PEG proved to have rather similar *D*_n,DLS_ values as their solid counterparts in water, demonstrating that the hydrophilic hollow HA-MIP/CP microparticles have similar spherical morphologies as their solid counterparts in water.

**Figure 2:**
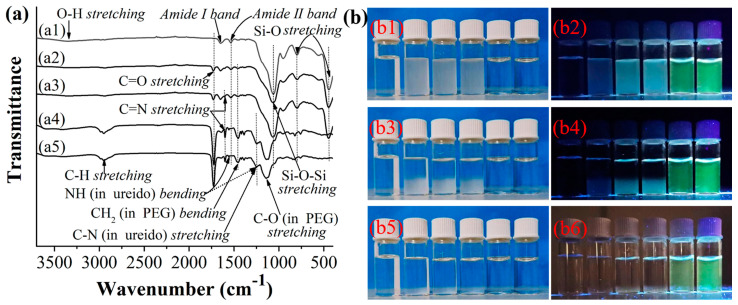
(**a**) FT-IR spectra of SiO_2_-Br (**a1**), SiO_2_@NBD-MIP@PEG (**a2**), SiO_2_@NBD-CP@PEG (**a3**), H@NBD-MIP@PEG (**a4**), and H@NBD-CP@PEG (**a5**). (**b**) Photographs of the ultrasonically dispersed aqueous mixtures (2.0 mg/mL; the concentration of the hollow samples was calculated by using the weights of their solid counterparts before etching) after being settled down at 20 °C for 0 h (**b1,b2**), 20 h (**b3,b4**), and 34 h (**b5,b6**), respectively. The samples located from left to right in each photograph are pure water, SiO_2_-Br, SiO_2_@NBD-MIP@PEG, SiO_2_@NBD-CP@PEG, H@NBD-MIP@PEG, and H@NBD-CP@PEG [the photographs of the aqueous mixtures were taken under the irradiation of the natural light (**b1,b3,b5**) and 365 nm UV light (**b2,b4,b6**)].

Figure 2a presents the FT-IR spectra of SiO_2_-Br and both the hydrophilic fluorescent solid and hollow HA-MIPs/CPs. The presence of the characteristic amide bands around 1647 cm^−1^ (amide I band) and 1535 cm^−1^ (amide II band) in the spectrum of SiO_2_-Br indicated that it had ATRP-initiating groups on the surface (Figure 2(a1)). Poly(EGDMA) and poly(4-VP) (and thus the MIP/CP layers) proved to be present onto the SiO_2_@NBD-MIP/CP@PEG surfaces because some new absorption bands (i.e., C=O stretching band around 1730 cm^−1^ and C=N stretching band around 1602 cm^−1^) appeared in their spectra in comparison with that of SiO_2_-Br (Figure 2(a2,a3)). In addition, the presence of PEG brushes on SiO_2_@NBD-MIP/CP@PEG was also verified by the existence of the new CH_2_ bending vibration band around 1460 cm^−1^ in their spectra compared with that of SiO_2_-Br. The successful syntheses of the hydrophilic fluorescent hollow HA-MIP/CP were verified by the disappearance of the Si-O-Si stretching peak around 1065 cm^−1^ and Si-O stretching peaks around 800 and 446 cm^−1^ in their spectra (Figure 2(a4,a5)). Note that the absorption bands of the NBD unit were not discernible in the spectra of the fluorescent solid HA-MIP/CP, mainly because of their overlap with those of the SiO_2_ core. This was confirmed by the appearance of the NBD absorption peaks around 1556 and 1252 cm^−1^ (stemming from the ureido group of NBD) in the spectra of H@NBD-MIP/CP@PEG. Moreover, the C-O (in PEG) stretching band around 1132 cm^−1^ (previously overlapped with the Si-O-Si stretching band of SiO_2_@NBD-MIP/CP@PEG) could also be clearly observed in the spectra of H@NBD-MIP/CP@PEG.

The static water contact angles of the films prepared with SiO_2_-Br and SiO_2_@NBD-MIP/CP@PEG were then measured to evaluate their surface hydrophilicity. SiO_2_@NBD-MIP/CP@PEG films showed reduced static water contact angles compared with the SiO_2_-Br film (entries 1–3, Table 1), which indicated that they were successfully grafted with the rather hydrophilic PEG brushes. Note that the static water contact angles of the H@NBD-MIP/CP@PEG films were not determined because only rather small amounts of the hollow HA-MIP/CP microparticles could be obtained after HF etching.

The aqueous dispersion stability of SiO_2_-Br and both the solid and hollow HA-MIP/CP was studied by monitoring the sedimentation processes of their ultrasonically dispersed mixtures in pure water (Figure 2b and Figure S2). It can be seen that SiO_2_-Br and the solid HA-MIP/CP formed white suspensions in pure water under the natural light irradiation (Figure 2(b1) and Figure S2(a1)). However, they showed a hyacinthine color (probably stemming from the scattering light of the 365 nm UV light) and cyan color (possibly stemming from a mixed color of the green fluorescence and the scattered 365 nm UV light), respectively, under the 365 nm UV light irradiation (Figure 2(b2) and Figure S2(a2)). Interestingly, the suspensions of the hollow HA-MIP/CP in pure water were found to be totally transparent under the natural light irradiation (they showed green fluorescence under the 365 nm UV light irradiation) (Figure 2(b1,b2) and Figure S2(a1,a2)), probably because of their ultrathin MIP/CP layers and very hydrophilic PEG brushes. The solid HA-MIP and CP showed almost the same (and rather slow) sedimentation speed as SiO_2_-Br although relatively larger static water contact angle was observed for the SiO_2_-Br film in comparison with the solid HA-MIP/CP films (entries 1–3, Table 1), which could be attributed to the relatively larger diameters of the solid HA-MIP/CP compared with SiO_2_-Br. These results again clearly demonstrated that the HA-MIP/CP layers with hydrophilic polymer brushes were successfully grafted onto the silica particles via the one-pot SI-ATRP. Note that, while the solid HA-MIP/CP fully settled to the bottom of the bottle in pure water after 34 h (Figure 2(b5) and Figure S2(h1)), the aqueous suspension of the hollow HA-MIP/CP remained transparent under the same condition and negligible sedimentation took place, as revealed by the still homogeneous green fluorescence of their aqueous suspensions (Figure 2(b5,b6) and Figure S2(h1,h2)). The above results strongly demonstrate that our hydrophilic fluorescent hollow HA-MIP/CP have largely improved aqueous dispersion stability compared with their solid counterparts, which is highly useful for their optosensing applications.

### 2.2. Equilibrium/Competitive Binding Properties of the Hydrophilic Fluorescent Solid and Hollow HA-MIPs/CPs in Different Media

Figure S3 shows the equilibrium template bindings of the hydrophilic fluorescent solid and hollow HA-MIPs/CPs in both the organic solvent (acetonitrile/methanol = 3:1 *v*/*v*) and artificial urine. Both the solid and hollow HA-MIPs exhibited obvious specific binding (i.e., the binding difference between the MIP and its CP [35]) in the organic solvent and artificial urine, indicating the existence of imprinted binding sites in these MIPs. In addition, the hydrophilic fluorescent solid HA-MIP showed a specific template binding in the artificial urine almost the same as it showed in the organic solvent (Figure S3a,b), mainly because of their high surface hydrophilicity (Table 1, Figure 2b) [21,22]. Similarly, the hydrophilic fluorescent hollow HA-MIP also exhibited good complex aqueous sample-compatiblity, as revealed by its presence of apparent specific template binding in the artificial urine. Nevertheless, it showed an obviously larger specific template binding in the artificial urine than in the organic solvent, which might stem from the somewhat different inner surfaces of the hollow HA-MIP and its CP, thus leading to the different template bindings on their inner surfaces.

The binding selectivity of the hydrophilic fluorescent solid and hollow HA-MIPs/CPs were then investigated by measuring their competitive bindings toward HA and its structural analogues (including 3-methylhippuric acid (3-MHA), 4-aminohippuric acid (4-AHA), and L-tyrosine (Tyr) (Scheme 1b)) in different media (Figure S4). Apparent HA selectivity was observed for both the solid and hollow HA-MIPs in both the organic solvent and artificial urine, as revealed by their much larger “imprinting-induced promotion of binding” (IPB) values toward HA than its analogues (Table S2) [36]. The above results clearly demonstrate that the combined use of one-pot SI-ATRP and the sacrificial template method is highly versatile for obtaining complex biological sample-compatible fluorescent hollow MIP microparticles.

### 2.3. Optosensing Properties of the Hydrophilic “Turn-On”-Type Fluorescent Solid and Hollow HA-MIP/CP Micropartilces in the Artificial Urine

**Figure 3:**
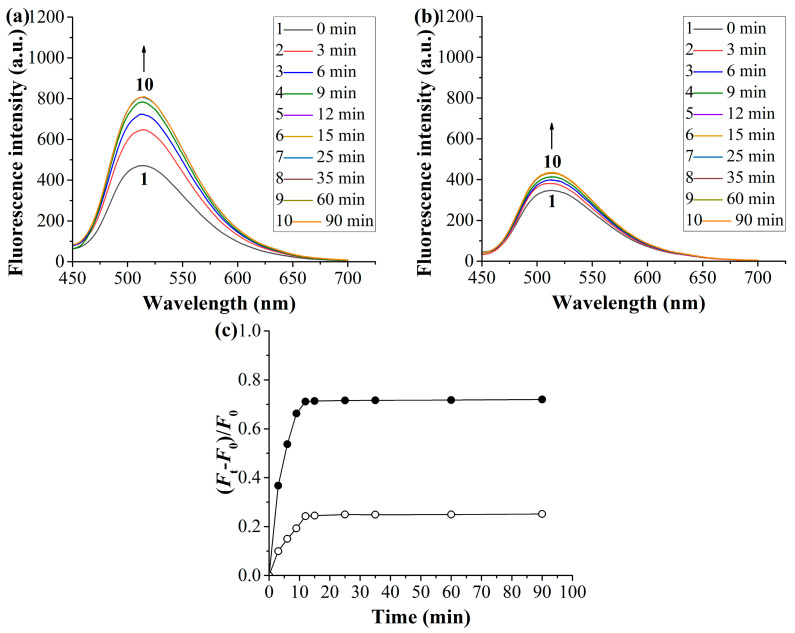
(**a**,**b**) Fluorescence spectra of the hydrophilic fluorescent hollow HA-MIP (**a**)/CP (**b**) after their incubation with a HA solution (20 μM) in the artificial urine at 25 °C for different times (hollow MIP/CP concentration: 0.25 mg/mL; their concentration was calculated by using the weight of their corresponding solid ones before etching). (**c**) Optosensing kinetics of the hydrophilic fluorescent hollow HA-MIP (filled symbol)/CP (open symbol) in a HA solution (20 μM) in the artificial urine at 25 °C (derived from Figure 3a,b; *F_t_* and *F*_0_ in (*F_t_* − *F*_0_)/*F*_0_ are the fluorescence intensity of the NBD unit (at 514 nm) at a time of *t* and 0, respectively).

In this section, the optosensing properties of the above-obtained hydrophilic fluorescent solid and hollow HA-MIP/CP microparticles in the artificial urine were investigated. We first studied their optosensing kinetics by recording the fluorescence spectra of their mixed solutions with HA in the artificial urine after being incubated for different times (Figure 3 and Figure S5). Both the hydrophilic fluorescent solid and hollow HA-MIPs/CPs showed “turn-on” fluorescence upon their exposure to HA solutions, and the fluorescence intensities of their NBD units (λ_max_ = 514 nm) were found to increase with time and then leveled off after 30 and 12 min, respectively. The above results indicated that the hydrophilic fluorescent hollow HA-MIP/CP had much faster optosensing kinetics than their solid counterparts in the artificial urine, just as observed by others for the (fluorescent) hollow MIPs in the organic solvents or mixtures of an organic solvent and water [29,30,31]. In addition, much larger fluorescence enhancement effect was observed for the fluorescent solid and hollow HA-MIPs than their corresponding HA-CPs, which could be ascribed to the existence of HA-imprinted binding sites in these HA-MIPs. The fluorescence enhancement of the fluorescent solid and hollow HA-MIPs was induced by both the stronger specific interaction between the imprinted binding sites and HA and the weaker nonspecific interaction between the MIP surfaces and HA. In contrast, the fluorescent solid and hollow HA-CPs only had weaker nonspecific interaction with HA, thus leading to their smaller fluorescence enhancement.

**Figure 4:**
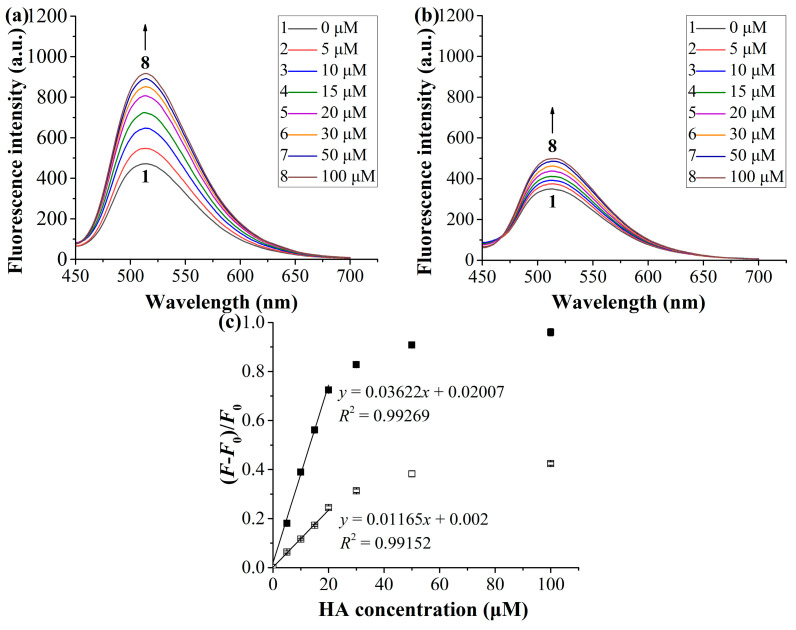
(**a**,**b**) Fluorescence spectra of the hydrophilic fluorescent hollow HA-MIP (**a**)/CP (**b**) upon their exposure to different concentrations of HA in the artificial urine at 25 °C for 2 h (hollow MIP/CP concentration: 0.25 mg/mL; their concentration was calculated by using the weights of their solid counterparts before etching). (**c**) Dependence of the fluorescence enhancement [(*F* − *F*_0_)/*F*_0_, where *F* and *F*_0_ are the fluorescence intensity of NBD fluorophores at 514 nm in the presence and absence of HA, respectively] of the hydrophilic fluorescent hollow HA-MIP (filled symbol)/CP (open symbol) on the HA concentration (derived from Figure 4a,b).

Some important optosensing parameters of the hydrophilic fluorescent solid and hollow HA-MIPs/CPs (including the linear detection range, limit of detection (LOD), and imprinting factor (IF)) were then determined by carrying out their spectrofluorimetric titration in the artificial urine (Figure 4 and Figure S6). The fluorescence intensities of both the hydrophilic fluorescent solid and hollow HA-MIPs/CPs proved to increase with an increase in the HA concentrations. In addition, both the hydrophilic fluorescent solid and hollow MIPs also exhibited much larger fluorescence enhancement effect than their corresponding CPs, just as observed in the above optosensing kinetic studies. By fitting these fluorescent titration results with the equation *F*/*F*_0_ = 1 + *KC* (where *F*_0_ and *F* are the fluorescence intensities in the absence and presence of HA, respectively, *K* is the constant, and *C* is the HA concentration) (Figure 4c and Figure S6c), linear calibration curves were achieved for the fluorescent solid and hollow HA-MIP chemosensors in the range of 0–20 μM. Moreover, the LOD values of the fluorescent solid and hollow HA-MIPs were determined to be 0.145 μM and 0.097 μM, respectively, by using the equation LOD = 3*δ*/*K*_MIP_ (where *δ* is the standard deviation of the blank measurements (for 20 times) and *K*_MIP_ is the slope of the linear optosensing calibration curves for the fluorescent solid and hollow HA-MIPs) [37]. It is noteworthy that the fluorescent hollow HA-MIP showed higher optosensing sensitivity than its corresponding solid HA-MIP, which could be attributed to the combined effect of its larger template binding capacity in the artificial urine (resulting in somewhat larger *K*_MIP_) and the higher aqueous dispersion stability (leading to smaller *δ*) than the solid HA-MIP. Furthermore, the IF values of the fluorescent solid and hollow HA-MIPs were derived to be 3.34 and 3.11 in the artificial urine, respectively, by using the equation IF = *K*_MIP_/*K*_CP_, which again confirmed that both the fluorescent solid and hollow HA-MIPs had HA-imprinted binding sites and high template recognition ability in complex aqueous media. Moreover, almost the same IF values of the fluorescent solid and hollow HA-MIPs indicated that the silica core-etching process hardly had a negative effect on the imprinted binding sites of the resulting fluorescent hollow HA-MIP. Based on the above optosensing results, we can conclude that the introduction of hollow cavities inside the hydrophilic fluorescent HA-MIP microparticles can result in much faster optosensing kinetics and higher optosensing sensitivity, which are highly useful for their real-world sensing applications.

**Figure 5:**
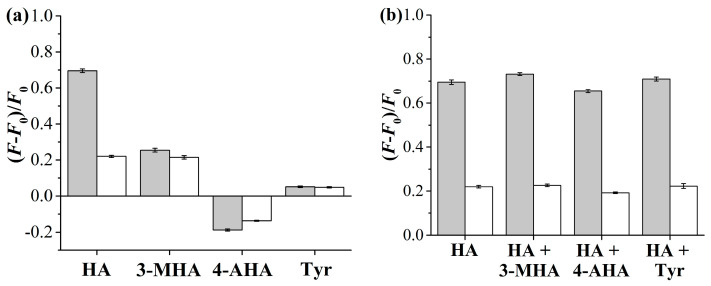
Fluorescence enhancement of the hydrophilic fluorescent hollow HA-MIP (filled column)/CP (open column) upon exposure to a HA, 3-MHA, 4-AHA, or Tyr solution (*C*_HA, 3-MHA, 4-AHA, or Tyr_ = 20 μM) (**a**) or to a HA solution (20 μM) in the presence of 40 μM of 3-MHA, 4-AHA, or Tyr (**b**) in the artificial urine at 25 °C for 2 h (hollow MIP/CP concentration: 0.25 mg/mL; their concentration was calculated by using the weights of their solid counterparts before etching).

The optosensing selectivity of the hydrophilic fluorescent solid and hollow HA-MIPs/CPs was also studied by exposing them to HA and its structural analogues [including 3-MHA, 4-AHA, and Tyr (Scheme 1b)] in the artificial urine. It can be seen from Figure 5 and Figure S7 that both the fluorescent solid and hollow HA-MIPs/CPs showed fluorescence enhancement toward 3-MHA and Tyr but fluorescence quenching toward 4-AHA. The fluorescence quenching of the studied HA-MIPs/CPs toward 4-AHA might be attributed to the electron transfer from the amino unit of 4-AHA to their embedded NBD units, just as described in a previous report by Chen and coworkers [38]. Nevertheless, the fluorescent solid and hollow HA-MIPs showed much larger fluorescence change toward HA than its analogues. In addition, the solid and hollow HA-MIPs/CPs exhibited almost the same and rather small fluorescence change toward all the analogues of HA. These results, together with the hardly changed fluorescence enhancement of these solid and hollow HA-MIPs even with the addition of two equivalent of its one analogue in the studied HA solutions, strongly demonstrate that both the hydrophilic fluorescent solid and hollow HA-MIPs have excellent optosensing selectivity toward HA in the artificial urine and their fluorescence enhancement toward the analogues of HA should be ascribed to nonspecific bindings.

Finally, the fluorescent stability and reusability of the hydrophilic fluorescent solid and hollow HA-MIPs/CPs were evaluated owing to their high importance for the real-world applications. Both the fluorescent solid and hollow HA-MIPs/CPs proved to have high photostability, as revealed by their negligible fluorescence intensity change around 514 nm after being put in pure water at room temperature under air atmosphere for 10 days (Figure S8). In addition, their excellent reusability was also clearly demonstrated by their nearly constant fluorescent intensities during 10 regeneration cycles (Figure S9).

### 2.4. Direct, Selective, Rapid, and Accurate Quantification of HA in the Undiluted Human Urine with the Hydrophilic “Turn-On”-Type Fluorescent Hollow HA-MIP

The hydrophilic “turn-on”-type fluorescent hollow HA-MIP was then used for direct and highly selective optosensing of HA in the undiluted human urine to demonstrate its real complex biological sample-compatibility. The presence of a certain amount of HA in the studied blank human urine sample was first confirmed by HPLC measurement, and the HA content was determined to be 1.56 μM (the human urine sample was pretreated prior to the HPLC analysis to remove proteins by adding methanol into it following the previously reported method [39,40]). Importantly, our hydrophilic fluorescent hollow HA-MIP optosensor also provided a rather close HA content for the blank human urine sample (i.e., 1.53 μM), which was rapidly obtained by directly measuring the fluorescence intensity of the incubated mixture of our fluorescent hollow HA-MIP and urine sample (after an incubation time of 15 min) and fitting the datum into the calibration curve achieved from the artificial urine sample optosensing.

With the above results in hand, we further detected the HA contents in the undiluted human urine samples spiked with different amounts of HA or a mixture of HA and its several analogues by using our hydrophilic fluorescent hollow HA-MIP. Good recoveries (96.0–102.0%) and low relative standard deviations (RSDs) (≤4.0%) were achieved for HA optosensing in all cases (entries 2–7, Table 2). In particular, these HA optosensing results agreed well with the HPLC characterization data (Table 2). Based on the above results, we can conclude that our hydrophilic “turn-on”-type fluorescent hollow HA-MIP is highly promising for direct, highly selective, rapid, and accurate HA optosensing in the complex biological samples without requiring any sample pretreatment and expensive instrument.

It is noteworthy here that our fluorescent hollow MIP optosensor shows apparent advantages over the previously reported MIP-based HA-detecting systems (Table 3) because no complex and tedious sample pretreatment is required during its optosensing complex biological samples. This, together with its high enough analytical sensitivity (its LOD value is much lower than the normally found HA levels in the healthy human urines (from several to dozens of μM [41,42] to several mM [43])) and prominent optosensing selectivity and accuracy, makes it highly promising in the practical bioanalytical and diagnostic applications (note that the HA contents in the urine samples can be easily measured through their dilution with water when their HA contents are beyond the linear range of our MIP optosensor).

## 3. Materials and Methods

### 3.1. Materials and Reagents

The materials and reagents utilized in this work (including the purification of copper(I) chloride (CuCl) [48] and preparation of 3-(*N*-propyl)triethoxysilane 2-bromo-2-methylpropanamide (BIBAPTES) [49], tris(2-(dimethylamino)ethyl)amine (Me_6_TREN) [50], 2-(3-(4-nitrobenzo[c][1,2,5]oxadiazo-7-yl)ureido)ethyl methacrylate (MA-Urea-NBD) [51], and artificial urine [52]) are described in the Supporting Information.

### 3.2. Preparation of the Core-Shell-Corona-Structured “Turn-On”-Type Fluorescent HA-Imprinted Polymer (HA-MIP)/Control Polymer (CP) (or Non-Imprinted Polymer) Microspheres with Labeled Fluorescent Nitrobenzoxadiazole (NBD) Unit and Polyethylene Glycol (PEG) Brushes [Briefly Hydrophilic Fluorescent Solid HA-MIP/CP (i.e., SiO_2_@NBD-MIP@PEG and SiO_2_@NBD-CP@PEG, Entries 2 and 3 in Table 1)]

The hydrophilic fluorescent solid HA-MIP microspheres (i.e., SiO_2_@NBD-MIP@PEG) were prepared via one-pot SI-ATRP in the presence of a hydrophilic macro-ATRP initiator [i.e., PEG with one alkyl bromide end-group (or ATRP-initiating group) (PEG-Br)] with the “living” silica particles (SiO_2_-Br, entry 1 in Table 1) (see their preparation in the Supporting Information) as the immobilized ATRP initiator following our previously reported procedure but with some modification (including the template and ratios of some reagents) [19]: 4-vinylpyridine (4-VP) (1.61 mmol), HA (0.81 mmol), MA-Urea-NBD (0.11 mmol), dried acetonitrile (70 mL), and methanol (24 mL) were added into a one-neck round-bottom flask (250 mL) with a magnetic stir bar inside successively. The self-assembly of the functional monomers and template was then carried out by first stirring the above solution in an ice-water bath for 2 h and then putting it in a refrigerator (4 °C) overnight. Afterwards, ethylene glycol dimethacrylate (EGDMA) (4.68 mmol) and Me_6_TREN (0.17 mmol) were added successively into the reaction system under stirring. After the above reaction mixture was bubbled with argon for 15 min in an ice-water bath, CuCl (0.057 mmol) was added. After another 15 min of argon bubbling through the reaction mixture, the “living” SiO_2_-Br particles (140.0 mg) and PEG-Br (116.2 mg) were added. The reaction system was then bubbled with argon for 5 min, sealed, and magnetically stirred (300 rpm) at 70 °C for 48 h. The product was collected by centrifugation, washed with a mixture of methanol and acetic acid (9:1 *v*/*v*) thoroughly (to remove the template) and then methanol, and finally dried at 40 °C under vacuum to a constant weight, leading to the desired SiO_2_@NBD-MIP@PEG with a weight increase of 18.4% compared with the starting “living” SiO_2_-Br particles (entry 2, Table 1).

The corresponding hydrophilic fluorescent solid CP microspheres (i.e., SiO_2_@NBD-CP@PEG) were also prepared and purified under the identical conditions, except for omitting HA. They showed a weight increase of 17.5% compared with the starting “living” SiO_2_-Br particles (entry 3, Table 1).

### 3.3. Preparation of the “Turn-On”-Type Fluorescent Hollow HA-MIP/CP Microparticles with PEG Brushes [Briefly Hydrophilic Fluorescent Hollow HA-MIP/CP (i.e., H@NBD-MIP@PEG and H@NBD-CP@PEG, Entries 4 and 5 in Table 1)]

The hydrophilic fluorescent hollow HA-MIP/CP microparticles (i.e., H@NBD-MIP/CP@PEG) were prepared by removing the silica core from their corresponding solid HA-MIP/CP microspheres (i.e., SiO_2_@NBD-MIP/CP@PEG) via hydrofluoric acid (HF) etching as follows: a dispersed suspension of SiO_2_@NBD-MIP/CP@PEG (0.25 mg/mL) in a mixture of HF aqueous solution (40%) and anhydrous ethanol (1:3 *v*/*v*) was incubated at 25 °C for 15 min, the resulting hollow polymer particles were collected by centrifugation, washed with methanol thrice, and then dried at 40 °C under vacuum to a constant weight, leading to H@NBD-MIP@PEG and H@NBD-CP@PEG with a weight decrease of 83.4% and 83.8% compared with SiO_2_@NBD-MIP@PEG and SiO_2_@NBD-CP@PEG, respectively (Table 1).

It is worth mentioning here that the hollow HA-MIP/CP became a hard sheet (or plate) after being dried under vacuum, which makes it rather difficult to be dispersed homogeneously in different solvents. Therefore, for analyzing the hollow HA-MIP/CP with different techniques and methods, the resulting H@NBD-MIP/CP@PEG (after etching their solid counterparts with HF in the solutions) were collected by centrifugation, washed with methanol and the corresponding solvent used for different analyses successively, and finally added into the respective solvent for different analyses such as atomic force microscope (AFM) and FT-IR characterization (methanol was used as the dispersing solvent, which was evaporated to dryness prior to analyses), dynamic light scattering (DLS) measurements (in the distilled water), dispersion stability test (in pure water), equilibrium/competitive binding studies (in the organic solvent (acetonitrile/methanol = 3:1 *v*/*v*) and artificial urine), and optosensing assays (in the artificial urine and undiluted human urine).

### 3.4. Characterization

The samples were characterized with ^1^H NMR spectrometer, FT-IR spectrometer, AFM [53], and DLS. The details of the above instruments and characterization are included in the Supporting Information.

The detailed information for studying the static water contact angles and aqueous dispersion stability of the samples and the equilibrium/competitive binding properties of the hydrophilic fluorescent solid and hollow HA-MIPs/CPs in different media (including the organic solvent and the artificial urine) is also presented in the Supporting Information.

The optosensing properties (including the optosensing kinetics and spectrofluorimetric titration in the artificial urine, photostability and reusability, and HA optosensing in the undiluted human urine) of the hydrophilic fluorescent solid and hollow HA-MIP/CP microparticles were characterized with an F-4600 spectrofluorometer (Hitachi, Japan). The excitation wavelength used was 420 nm, the voltage was 600 V, and the slit width of both the excitation and emission was 10 nm. The fluorescence intensities of NBD fluorophores around 514 nm were selected for the optosensing analyses.

## 4. Conclusions

We have demonstrated for the first time the development of hydrophilic “turn-on”-type fluorescent hollow MIP microparticles with highly efficient optosensing capability toward HA in the undiluted human urine. They were readily obtained through first the controlled grafting of a green NBD-labeled fluorescent ultrathin HA-MIP layer with hydrophilic polymer brushes onto the preformed “living” silica particles via one-pot SI-ATRP and their subsequent removal of the silica core via HF etching. They proved to show outstanding optosensing selectivity and sensitivity toward HA as well as prominent photostability and reusability. More importantly, their hollow cavities endowed them with significant advantages over their solid counterparts including much more stable aqueous dispersion ability, dramatically faster optosensing speed, and higher optosensing sensitivity, which are highly useful for practical optosensing applications. Their direct, highly selective, rapid, and accurate quantification of HA in the undiluted human urine samples was also confirmed. We believe that such advanced complex biological sample-compatible fluorescent hollow MIP microparticles are of great potential in many real-world bioanalyses and clinical diagnoses.

## Figures and Tables

**Table 1:** Synthetic and characterization data of the “living” silica particles and both the hydrophilic fluorescent solid and hollow HA-MIP/CP particles.

Entry	Sample	Δ*W* (%) ^a^	*D*_n,AFM_ (nm) ^b^	*U* ^b^	*D*_n,DLS_ (nm) ^c^	PDI ^c^	Contact Angle (°) ^d^
1	SiO_2_-Br	-	496	1.004	529	0.112	78.2 ± 2.6
2	SiO_2_@NBD-MIP@PEG	18.4	525	1.005	573	0.134	64.6 ± 2.7
3	SiO_2_@NBD-CP@PEG	17.5	523	1.010	570	0.115	64.9 ± 2.1
4	H@NBD-MIP@PEG	83.4	-	-	568	0.129	-
5	H@NBD-CP@PEG	83.8	-	-	566	0.158	-

^a^ The enhanced weight percentage of SiO_2_@NBD-MIP/CP@PEG compared with the starting SiO_2_-Br and reduced weight percentage of H@NBD-MIP/CP@PEG compared with SiO_2_@NBD-MIP/CP@PEG. ^b^ *D*_n,AFM_ and *U* refer to the number-average diameter and size distribution index of the samples determined by AFM, respectively. ^c^ *D*_n,DLS_ and PDI denote the number-average hydrodynamic diameter and particle dispersion index of the samples determined by DLS in water, respectively. ^d^ The static water contact angles of the sample films.

**Table 2:** Direct detection of HA in the undiluted urine samples at three spiking levels of both HA and its mixtures with several analogues with the hydrophilic fluorescent hollow HA-MIP ^a^.

Entry	Analyte(s)	Spiked Analyte(s) (μM)	Detected by MIP Optosensor HA (μM)	Optosensing Recovery ± RSD (%) (*n* = 3) ^b^	Detected by HPLC HA (μM)	HPLC Recovery ± RSD (%) (*n* = 3) ^b,c^
1	HA	0 (Blank urine)	1.53	-	1.56	-
2	HA	0.5	2.01 ± 0.02	96.0 ± 4.0	2.08 ± 0.02	103.7 ± 4.4
3	HA	5	6.52 ± 0.06	99.8 ± 1.2	6.61 ± 0.09	101.0 ± 1.8
4	HA	10	11.64 ± 0.06	101.1 ± 0.6	11.50 ± 0.15	99.4 ± 1.5
5	HA + 3-MHA + 4-AHA + Tyr	0.5 HA + 0.5(3-MHA) + 0.5(4-AHA) + 0.5 Tyr	2.02 ± 0.01	98.0 ± 2.0	2.07 ± 0.02	102.3 ± 4.1
6	HA + 3-MHA + 4-AHA + Tyr	5 HA + 5(3-MHA) + 5(4-AHA) + 5 Tyr	6.63 ± 0.08	102.0 ± 1.6	6.74 ± 0.07	103.5 ± 1.5
7	HA + 3-MHA + 4-AHA + Tyr	10 HA + 10(3-MHA) + 10(4-AHA) + 10 Tyr	11.69 ± 0.09	101.6 ± 0.9	11.62 ± 0.23	100.6 ± 2.3

^a^ HA optosensing was carried out by first incubating a mixture of the fluorescent hollow HA-MIP (0.25 mg/mL, the concentration of the hollow MIP was calculated by using the weight of its solid counterpart before its etching) and the undiluted human urine samples without or with spiked analytes for 15 min and then directly measuring their fluorescent spectra. ^b^ These optosensing and HPLC recoveries were derived by excluding the initial HA concentration in the urine samples determined by the hollow MIP optosensor (1.53 μM) and HPLC (1.56 μM), respectively. ^c^ All human urine samples were pretreated to remove proteins by first adding methanol into them (methanol/urine = 1:1 *v*/*v*) and then centrifugating the samples (10,000 rpm) for 10 min prior to HPLC analyses [39,40].

**Table 3:** Performance comparison of our hydrophilic fluorescent hollow HA-MIP optosensor with other previously reported MIP-based detection systems for HA.

Analytical Method ^a^	Sample	Linear Range	LOD	Recovery (%)	RSD (%)	Ref.
Solid-phase extraction (SPE)/HPLC-UV	Human urine (filtered through Whatman paper No. 42)	0.3–7500 μg/L (0.0017–41.86 μM)	0.15 μg/L (0.84 nM)	88.0–104.0	<6.1	[44]
SPE/LC-MS/MS	Human urine (filtered through a 0.22 μm PTFE membrane)	0.5–10,000 μg/L (0.0028–55.81 µM)	89 ng/L (0.50 nM)	91.4–109.1	6.4–9.6 (intra-day) 9.2–11.5 (inter-day)	[41]
SPE/micellar electrokinetic chromatography (MEKC)	Human urine (without pretreatment)	0.5–5.0 g/L (2.79–27.91 mM)	0.15 g/L (0.84 mM)	-	<16	[45]
Micro-extraction by packed sorbent (MEPS)/LC-MS/MS	Plasma and urine (pretreated to remove proteins with acetonitrile)	1–1000 nM	0.3 nM	91–96	1.1–7.1	[46]
Hollow fiber based liquid-phase microextraction/ LC-MS/MS	Human plasma and urine [pretreated to remove proteins with 25 mM ammonium acetate (pH 5.0)]	1–2000 nM	0.3 nM	97–104	1.2–4.1	[33]
Electrochemical sensing	Human serum (pretreated to remove proteins with methanol) and diluted human urine	0.05–40 nM and 40–500 nM	0.012 nM	96.0–105.0	1.2–3.2	[47]
Direct fluorescent optosensing	Human urine (without any pretreatment)	0–20 µM	0.097 µM	96.0–102.0	0.6–4.0	This work

^a^ SPE separation normally requires four steps (i.e., column condition, sample upload, wash, and elution), which is rather time-consuming and tedious. In addition, many SPE parameters (e.g., the sample pH, the amount of the sorbent, and the washing and elution solvent volumes) also need optimization prior to the sample extraction.

## Data Availability

Not applicable.

## Supplementary Materials

The following supporting information can be downloaded at: https://www.mdpi.com/article/10.3390/molecules28031077/s1, Figure S1: Fluorescence spectra of MA-Urea-NBD (2.5 mM) after its incubation with different concentrations of HA in acetonitrile/methanol (3:1 *v*/*v*) at 25 °C for 2 h; Figure S2: Detailed photographs of the ultrasonically dispersed aqueous mixtures (2.0 mg/mL, the concentration of the hollow samples was calculated by using the weights of their solid counterparts before etching) after being settled down at 20 °C for 0 h (a1,a2), 6 h (b1,b2), 10 h (c1,c2), 16 h (d1,d2), 20 h (e1,e2), 24 h (f1,f2), 30 h (g1,g2), and 34 h (h1,h2), respectively. The samples located from left to right in each photograph are pure water, SiO_2_-Br, SiO_2_@NBD-MIP@PEG, SiO_2_@NBD-CP@PEG, H@NBD-MIP@PEG, and H@NBD-CP@PEG [the photographs of the aqueous mixtures were taken under the irradiation of the natural light (a1-h1) and 365 nm UV light (a2-h2)]; Figure S3: Equilibrium bindings of HA on the hydrophilic fluorescent solid and hollow HA-MIPs/CPs in their solutions in acetonitrile/methanol (3:1 *v*/*v*) (a) and artificial urine (b) at 25 °C, respectively (*C*_0 HA_ = 0.01 mM; polymer concentration: 2 mg/mL; the concentration of the hollow samples was calculated by using the weights of their solid counterparts before etching); Figure S4: Competitive bindings of the hydrophilic fluorescent solid and hollow HA-MIPs/CPs toward HA, 3-MHA, 4-AHA, and Tyr in their mixed solutions in acetonitrile/methanol (3:1 *v*/*v*) (a) and artificial urine (b), respectively (*C*_0 HA or 3-MHA or 4-AHA or Tyr)_ = 0.01 mM; polymer concentration: 2 mg/mL; the concentration of the hollow samples was calculated by using the weights of their solid counterparts before etching); Figure S5: (a,b) Fluorescence spectra of the hydrophilic fluorescent solid HA-MIP (a)/CP (b) (0.25 mg/mL) after their incubation with a HA solution (20 μM) in the artificial urine at 25 °C for different times. (c) Optosensing kinetics of the hydrophilic fluorescent solid HA-MIP (filled symbol)/CP (open symbol) in a HA solution (20 μM) in the artificial urine at 25 °C [derived from Figure S5a,b; *F_t_* and *F*_0_ in (*F_t_* -*F*_0_)/*F*_0_ are the fluorescence intensity of the NBD unit (at 514 nm) at a time of *t* and 0, respectively]; Figure S6: (a,b) Fluorescence spectra of the hydrophilic fluorescent solid HA-MIP (a)/CP (b) upon their exposure to different concentrations of HA in the artificial urine at 25 °C for 2 h (MIP/CP concentration: 0.25 mg/mL). (c) Dependence of the fluorescence enhancement [(*F*-*F*_0_)/*F*_0_] of the hydrophilic fluorescent solid HA-MIP (filled symbol)/CP (open symbol) on the HA concentration (derived from Figure S6a,b); Figure S7: Fluorescence enhancement of the hydrophilic fluorescent solid HA-MIP (filled column)/CP (open column) upon exposure to a HA, 3-MHA, 4-AHA, or Tyr solution (*C*_HA, 3-MHA, 4-AHA, or Tyr_ = 20 μM) (a) or to a HA solution (20 μM) in the presence of 40 μM of 3-MHA, 4-AHA, or Tyr (b) in the artificial urine at 25 °C for 2 h (MIP/CP concentration: 0.25 mg/mL); Figure S8: The fluorescence intensity changes (around 514 nm) of dispersed mixtures of SiO_2_@NBD-MIP/CP@PEG (a) or H@NBD-MIP/CP@PEG (b) in pure water over time at 25 °C under air atmosphere (HA-MIP/CP concentration: 0.25 mg/mL, the concentration of the hollow HA-MIP/CP was calculated by using the weights of their solid counterparts before etching); Figure S9: Fluorescence intensity changes (around 514 nm) of SiO_2_@NBD-MIP/CP@PEG (a) or H@NBD-MIP/CP@PEG (b) upon desorption (empty) and adsorption (filled) of HA (20 μM) in the artificial urine during their 10 regeneration cycles (HA-MIP/CP concentration: 0.25 mg/mL, the concentration of the hollow HA-MIP/CP was calculated by using the weights of their solid counterparts before etching); Table S1: Synthetic and characterization data of two batches of “living” silica particles and hydrophilic fluorescent solid HA-MIP/CP particles; Table S2: Competitive binding properties of the hydrophilic fluorescent solid and hollow HA-MIPs/CPs toward HA and its analogues in different media.
