# Supplementary material for: Efficient Optosensing of Hippuric Acid in the Undiluted Human Urine with Hydrophilic “Turn-On”-Type Fluorescent Hollow Molecularly Imprinted Polymer Microparticles
Source: Molecules. 2023 Jan 20;28(3):1077. doi: 10.3390/molecules28031077 (PMC9920520; doi:10.3390/molecules28031077)
Supplement: Supplementary file 1 [file molecules-28-01077-s001.zip › molecules-2114803-supplementary.pdf]

Supplementary Information (SI)

Efficient Optosensing of Hippuric Acid in the Undiluted Human Urine with Hydrophilic "Turn-On"-Type Fluorescent Hollow Molecularly Imprinted Polymer Microparticles

Wanlan Zhang, Qun Li and Huiqi Zhang*

State Key Laboratory of Medicinal Chemical Biology, Key Laboratory of Functional Polymer Materials (Ministry of Education), Collaborative Innovation Center of Chemical Science and Engineering (Tianjin), Frontiers Science Center for New Organic Matter, and College of Chemistry, Nankai University, Tianjin 300071, China

Materials and reagents

4-Vinylpyridine (4-VP, Alfa Aesar, 96%) was purified by distillation under vacuum. Ethylene glycol dimethacrylate (EGDMA, Alfa Aesar, 98%) was washed with an aqueous solution of sodium hydroxide (10%) and water, dried with anhydrous magnesium sulfate, and then distilled under vacuum. Acetonitrile (Tianjin Concord Technology Co., Ltd., analytical reagent (AR)) was refluxed over calcium hydride and then distilled. Copper (I) chloride [CuCl, Meryer (Shanghai) Chemical Technology Co., Ltd., 99%] was purified according to the literature method [1]. 3-(*N*-Propyl)triethoxysilane 2-bromo-2-methylpropanamide (BIBAPTES) [2], tris(2-(dimethylamino)ethyl)amine (Me_6TREN) [3], and 2-(3-(4-nitrobenzo[c][1,2,5]oxadiazo-7-yl)ureido)ethyl methacrylate (MA-Urea-NBD) [4] were prepared following the literature approaches. The polyethylene glycol (PEG) macro-ATRP initiator (PEG-Br**)** was prepared by reacting the monomethoxy-capped PEG (MeO-PEG-OH, M_n = 5000 g/mol) and 2-bromoisobutyryl bromide (BIBB) with triethylamine as the catalyst according to our previous method and its successful synthesis was confirmed by ^1H NMR [5]. The artificial urine was prepared by dissolving urea (2.427 g), uric acid (0.034 g), creatinine (0.090 g), $Na_3C_6H_5O_7.2H_2O$ (0.297 g), NaCl (0.634 g), KCl (0.450 g), NH_4Cl (0.161 g), $CaCl_2.2H_2O$ (0.089 g), $MgSO_4.7H_2O$ (0.100 g), $NaHCO_3$ (0.034 g), NaC_2O_4 (0.003 g), Na_2SO_4 (0.258 g), $NaH_2PO_4.H_2O$ (0.100 g), and Na_2HPO_4 (0.011 g) in pure water (200 mL) following a literature method [6]. The obtained artificial urine was stored at 4 ^oC prior to use. The human urine was obtained from a young male volunteer

without exposure to toluene and used directly without any pretreatment. Hippuric acid (HA, Shanghai Xinbo Chemical Technology Co., Ltd., 98%), 3-methylhippuric acid (3-MHA, Shanghai Macklin Biochemical Technology Co., Ltd, ≥98%), 4-aminohippuric acid (4-AHA, Shanghai Macklin Biochemical Technology Co., Ltd, 98%), L-tyrosine [Tyr, Shanghai Macklin Biochemical Technology Co., Ltd, 99% (biotechnology grade)], tetraethylorthosilicate (TEOS, Shanghai Aladdin Bio-Chem Technology Co., LTD, 98%), and all the other reagents were commercially available and used as received. The chemical structures of some reagents mentioned above are presented in Scheme 1b.

Synthesis of uniform "living" silica microspheres bearing ATRP-initiating groups (i.e., SiO_2-Br, entry 1 in Table 1)

SiO_2-Br microspheres were prepared via one-pot sol-gel reaction of TEOS in the presence of BIBAPTES (Scheme 1b) following a literature method but with some modification [7]: TEOS (2.7 mL) was added dropwise into a mixture of pure water (6.5 mL), ethanol (48.0 mL), and an aqueous solution of ammonia (25%) (5.4 mL). The mixed solution was magnetically stirred (200 rpm) at 25 °C for 20 min, followed by the dropwise addition of APTES-Br (0.4 g) under stirring. The reaction mixture was then stirred at 25 °C for 10 h. The resulting SiO_2-Br particles were collected by centrifugation, washed thoroughly with distilled water until the washing solution became neutral, and then dried at 40 °C under vacuum to a constant weight. SiO_2-Br microspheres were obtained as a white solid powder in a yield of 25%.

Spectrofluorimetric tirtation of MA-Urea-NBD with HA

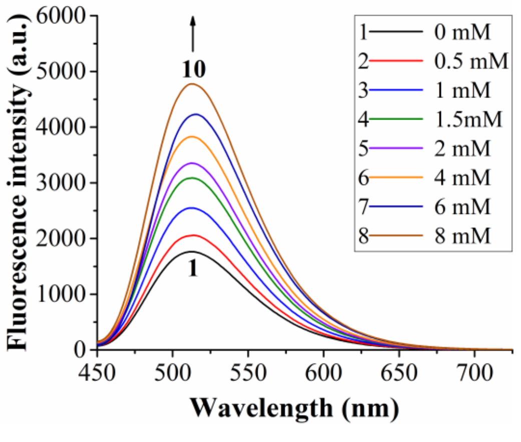

Figure S1. Fluorescence spectra of MA-Urea-NBD (2.5 mM) after its incubation with different concentrations of HA in acetonitrile/methanol (3:1 v/v) at 25 °C for 2 h.

AFM Characterization

The morphologies, particle sizes, and size distributions of the samples were characterized with a Dimension Icon (Bruker) atomic force microscope (AFM). The AFM tips are high-resolution noncontact "OTESPA" AFM probes with a 10 nm typical tip curvature radius. SiO_2-Br and hydrophilic fluorescent solid HA-MIP/CP (i.e., SiO_2@NBD-MIP/CP@PEG) dispersed in methanol (0.5 mg/mL) were dropped onto freshly cleaned micas, their topographic images were then acquired by the tapping measurement mode of the AFM after the samples were evaporated to dryness (Figure 1a-c). The diameters of the particles were evaluated from their AFM height profiles. All of the AFM size data reflected the averages of 200 particles, which were calculated by using the following formulas according to the literature (entries 1-3, Table 1) [8]:

$$D_n = \sum_{i=1}^{k} n_i D_i / \sum_{i=1}^{k} n_i; \quad D_w = \sum_{i=1}^{k} n_i D_i^4 / \sum_{i=1}^{k} n_i D_i^3; \quad U = D_w / D_n$$

where D_n is the number-average diameter, D_w the weight-average diameter, U the size distribution index, k the total number of the measured particles, D_i the diameter of the ith particle, and n_i the number of particles with a diameter D_i.

The hydrophilic fluorescent hollow HA-MIP/CP (i.e., H@NBD-MIP/CP@PEG) were first prepared by dispersing SiO_2@NBD-MIP/CP@PEG (0.25 mg/mL) in a mixed solution of hydrofluoric acid (HF) aqueous solution (40%) and anhydrous ethanol (1:3 v/v) at 25 °C for 15 min. The resulting H@NBD-MIP/CP@PEG were collected by centrifugation, washed with methanol thrice, and then dropped onto freshly cleaned micas. Their topographic images were then acquired by the tapping measurement mode of the AFM after the samples were evaporated to dryness (Figure 1d,e).

Dynamic light scattering (DLS) analyses

A DLS spectrometer (Zetasizer Nano S90) with a CROSS-PMT detector was also used to obtain the sizes and particle dispersion indices (PDIs) of the samples in the distilled water (0.05 mg/mL) (entries 1-5, Table 1). The scattered light of a vertically polarized He-Ne laser (633 nm) was measured at an angle of 90° at 20 ^{o}C.

FT-IR Characterization

Fourier transform infrared (FT-IR) spectra of the samples were obtained using a Bruker TENSOR II FT-IR spectrometer by using the attenuated total reflectance (ATR) method (Figure 2a).

Static water contact angle measurements

The static water contact angles of SiO_2-Br and SiO_2@NBD-MIP/CP@PEG (entries 1-3, Table 1) were studied as follows: The films of SiO_2-Br and SiO_2@NBD-MIP/CP@PEG were prepared by casting their ultrasonically dispersed suspension solutions in DMF (10 mg/mL) on clean glass surfaces. After the solvent was evaporated at ambient temperature overnight and the films were dried under vacuum at 40 ^oC for 2 days, an optical contact angle analyzer (Dongguan Shengding Precision Instrument Co., Ltd., China) was used to determine their static water contact angles. Three measurements were taken across each sample, with their average being used for analysis (entries 1-3, Table 1).

Aqueous dispersion stability test

The aqueous dispersion stability of SiO_2-Br, SiO_2@NBD-MIP/CP@PEG, and H@NBD-MIP/CP@PEG (entries 1-5, Table 1) were studied as follows: The samples were first ultrasonically dispersed in pure water (2 mg/mL, the concentration of H@NBD-MIP/CP@PEG was calculated by using the weight of their solid counterparts before etching) and the aqueous suspensions were then allowed to settle down for different times at 20 °C. Their photographs were taken under the irradiation of both the natural light and 365 nm UV light (mainly for observing the fluorescence of the aqueous suspensions of H@NBD-MIP/CP@PEG because they are fully transparent under the natural light irradiation) (Figures 2b and S2).

Equilibrium and competitive binding experiments with the hydrophilic fluorescent solid and hollow HA-MIPs/CPs in different media

The equilibrium binding properties of the hydrophilic fluorescent solid and hollow HA-MIPs/CPs were evaluated by incubating them (2 mg/mL, the concentration of the hollow samples was calculated by using the weights of their solid counterparts before etching) with

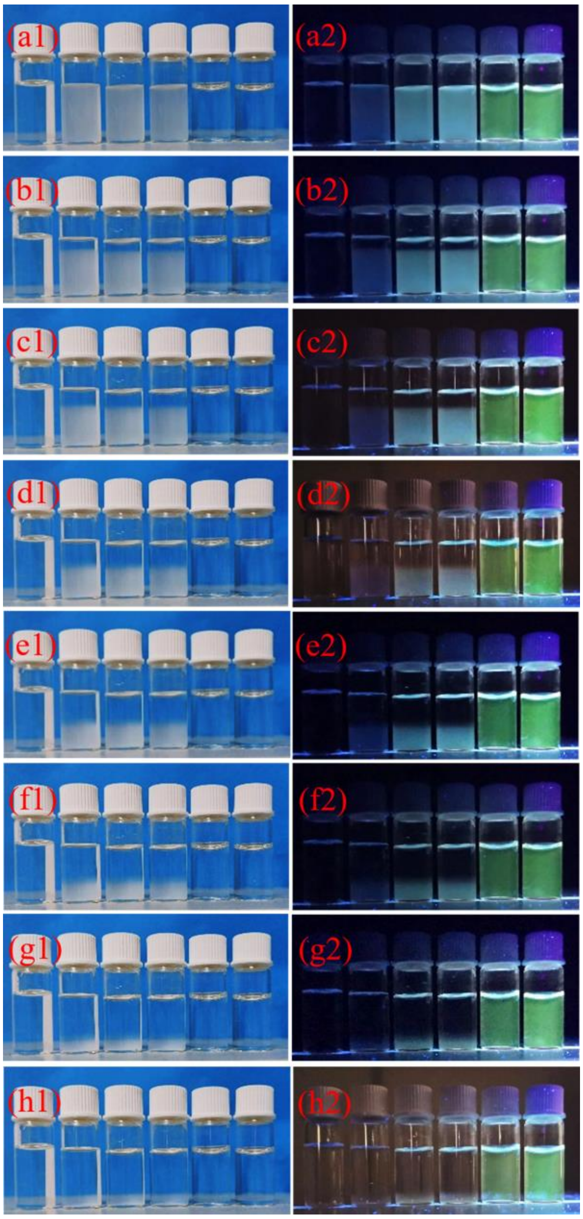

Figure S2. Detailed photographs of the ultrasonically dispersed aqueous mixtures (2.0 mg/mL, the concentration of the hollow samples was calculated by using the weights of their solid counterparts before etching) after being settled down at 20 ^{o}C for 0 h (a1,a2), 6 h (b1,b2), 10 h (c1,c2), 16 h (d1,d2), 20 h (e1,e2), 24 h (f1,f2), 30 h (g1,g2), and 34 h (h1,h2), respectively. The samples located from left to right in each photograph are pure water, SiO_2-Br, SiO_2@NBD-MIP@PEG, SiO_2@NBD-CP@PEG, H@NBD-MIP@PEG, and H@NBD-CP@PEG [the photographs of the aqueous mixtures were taken under the irradiation of the natural light (a1-h1) and 365 nm UV light (a2-h2)].

HA in different media [including the organic solvent (acetonitrile/methanol = 3:1 v/v) and the artificial urine] (C_0 = 0.01 mM, 0.5 mL) at 25 °C for 8 h, and the amounts of HA bound to MIPs/CPs [expressed as B (μmol/g)] were determined by measuring those remaining in the

solutions (expressed as F) with high performance liquid chromatography (HPLC, Scientific System Inc., USA) in conjunction with a UV-vis detector [i.e., $B = (C_0 - F)V/W$ (V is the volume of the used HA solution and W is the mass of the used polymer)]. The wavelength used for the determination of HA was 254 nm. A mixture of methanol, pure water, and acetic acid (25:74.85:0.15 v/v/v) was used as the mobile phase at a flow rate of 1 mL/min. All the above (and following) binding analyses were performed in triplicate and the mean values were used.

The competitive binding properties of the hydrophilic fluorescent solid and hollow HA-MIPs/CPs were analyzed by incubating them (2 mg/mL, the concentration of the hollow samples was calculated by using the weights of their solid counterparts before etching) with a mixture of HA and its analogues (including 3-MHA, 4-AHA, and Tyr, Scheme 1b) in both the organic solvent (acetonitrile/methanol = 3:1 v/v) and artificial urine (C_0 = 0.01 mM, 0.5 mL) at 25 °C for 8 h. The amounts of these analytes bound to MIPs/CPs were determined by measuring those remaining in the solutions with HPLC, following the similar procedures and methods as used in the equilibrium binding experiments.

Figure S3a,b presents the equilibrium template bindings of the hydrophilic fluorescent solid and hollow HA-MIPs/CPs in the organic solvent [acetonitrile/methanol (3:1 v/v)] and artificial urine, respectively. Both the solid and hollow HA-MIPs exhibited higher template

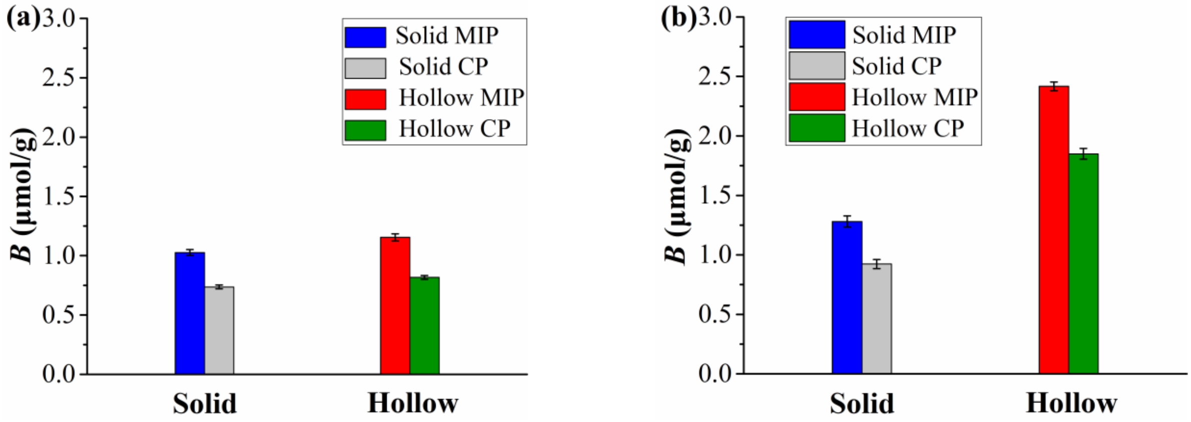

Figure S3. Equilibrium bindings of HA on the hydrophilic fluorescent solid and hollow HA-MIPs/CPs in their solutions in acetonitrile/methanol (3:1 v/v) (a) and artificial urine (b) at 25 ºC, respectively ($C_{0\,HA}$ = 0.01 mM; polymer concentration: 2 mg/mL; the concentration of the hollow samples was calculated by using the weights of their solid counterparts before etching).

binding capacities than their corresponding CPs in both the organic solvent and artificial urine (Figure S3), suggesting their presence of imprinted binding sites and their high complex aqueous sample-compatibility. Their high complex aqueous sample-compatibility can be attributed to their high surface hydrophilicity, as revealed by their low static water contact angle (Table 1) and high aqueous dispersion stability (Figures 2b and S2). Note that the hollow HA-MIP only showed slightly higher template binding capacity than its solid counterpart in the organic solvent (Figure S3a), which might be ascribed to the very thin MIP layer of the solid HA-MIP with most of its imprinted binding sites easily accessable. In addition, both the hollow HA-MIP and CP showed much higher template binding capacities than their solid counterparts in the artificial urine (Figure S3b), which might be due to the rather high surface hydrophobicity of the inner surfaces of the hollow HA-MIP/CP microparticles in the artificial urine, thus leading to high nonspecific template bindings. It is also noteworthy that the hollow HA-MIP showed larger specific template binding (i.e., the template binding difference between the MIP and its CP [9]) in the artificial urine than in the organic solvent, which might stem from the different inner surfaces of the hollow HA-MIP and its CP.

Figure S4a,b presents the competitive bindings of the hydrophilic fluorescent solid and hollow HA-MIPs/CPs toward HA and its structural analogues [including 3-MHA, 4-AHA, and Tyr (Scheme 1b)] in acetonitrile/methanol (3:1 v/v) and artificial urine, respectively, from which the "imprinting-induced promotion of binding" (IPB) values of the studied HA-MIPs were derived and listed in Table S1.

IPB is a useful parameter for evaluating the MIPs' selectivity because the difference in the intrinsic nonspecific bindings of MIPs toward different analytes is normalized [10]. IPB can be defined by the equation: IPB (%) = $[(B_{\mathrm{MIP}} - B_{\mathrm{CP}})/B_{\mathrm{CP}}] \times 100$, where B_{MIP} and B_{CP} are the equilibrium bindings of the studied MIP and its corresponding CP toward an analyte, respectively. The larger the IPB value of the MIP toward the analyte, the better the selectivity of the MIP. It can be seen from Table S1 that both the hydrophilic fluorescent solid and hollow HA-MIP showed obvious selectivity toward HA in both the organic solvent and artificial urine.

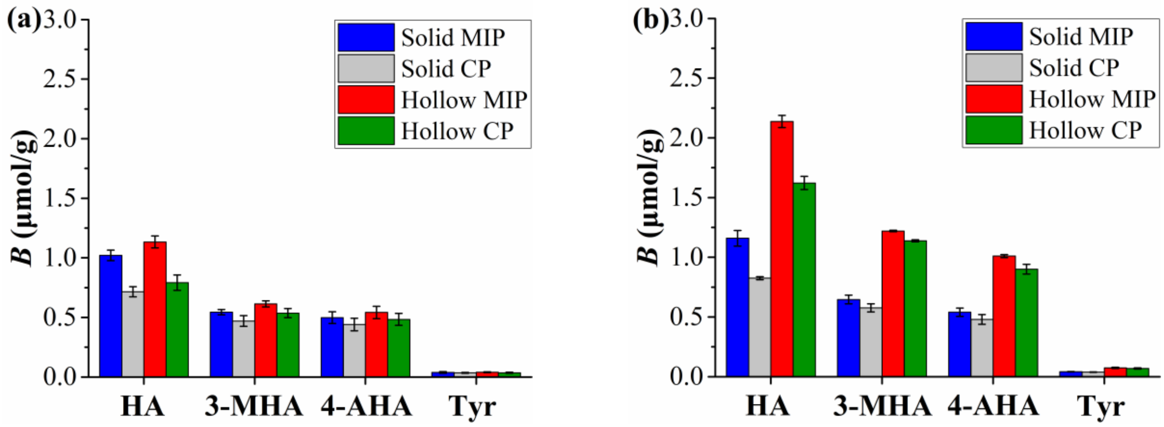

Figure S4. Competitive bindings of the hydrophilic fluorescent solid and hollow HA-MIPs/CPs toward HA, 3-MHA, 4-AHA, and Tyr in their mixed solutions in acetonitrile/methanol (3:1 v/v) (a) and artificial urine (b), respectively ($C_{0\ \mathrm{HA\ or\ 3\text{-}MHA\ or\ 4\text{-}AHA\ or\ Tyr}}$ = 0.01 mM; polymer concentration: 2 mg/mL; the concentration of the hollow samples was calculated by using the weights of their solid counterparts before etching).

Table S1. Competitive binding properties of the hydrophilic fluorescent solid and hollow HA-MIPs/CPs toward HA and its analogues in different media.

Solvent	Analyte	SiO_2@NBD-MIP/CP@PEG			H@NBD-MIP/CP@PEG		
		B_{MIP} [a]	B_{CP} [a]	**IPB** (%)	B_{MIP} [a]	B_{CP} [a]	**IPB** (%)
Acetonitrile /methanol (3:1 v/v)	HA	1.02±0.04	0.72±0.04	**42**	1.13±0.05	0.80±0.06	**41**
	3-MHA	0.54±0.02	0.48±0.05	**13**	0.61±0.03	0.54±0.04	**13**
	4-AHA	0.50±0.05	0.44±0.05	**14**	0.54±0.05	0.48±0.05	**13**
	Tyr	0.039±0.007	0.035±0.003	**11**	0.040±0.004	0.036±0.005	**11**
Artificial urine	HA	1.16±0.06	0.83±0.01	**40**	2.14±0.05	1.62±0.06	**32**
	3-MHA	0.65±0.04	0.58±0.04	**12**	1.22±0.01	1.14±0.01	**7**
	4-AHA	0.54±0.04	0.48±0.04	**13**	1.01±0.01	0.90±0.04	**12**
	Tyr	0.042±0.001	0.038±0.001	**11**	0.075±0.005	0.069±0.005	**9**

[a] B_{MIP} and B_{CP} are the equilibrium binding capacities of the studied MIPs and their CPs toward HA and its analogues in their mixed solutions ($C_{\mathrm{HA\ or\ 3\text{-}MHA\ or\ 4\text{-}AHA\ or\ Tyr}}$ = 0.01 mM) in different media, which are the same as those shown in Figure S4a,b and have a unit of μmol/g.

Optosensing properties of the hydrophilic fluorescent solid and hollow HA-MIP/CP microparticles

Optosensing kinetics of the hydrophilic fluorescent solid and hollow HA-MIPs/CPs in the artificial urine

The optosensing kinetics of the hydrophilic fluorescent solid and hollow HA-MIPs/CPs were studied by incubating a certain amount of MIP/CP (0.25 mg/mL, the concentration of the hollow samples was calculated by using the weights of their solid counterparts before etching) with a HA solution in the artificial urine (20 μM) for different times and their fluorescence spectra were recorded for analyses.

Figures 3a,b and S5a,b present the fluorescence spectra of the hydrophilic fluorescent hollow and solid HA-MIPs/CPs after their incubation with a HA solution 25 °C for different times in the artificial urine, from which their optosensing kinetics were derived (Figures 3c

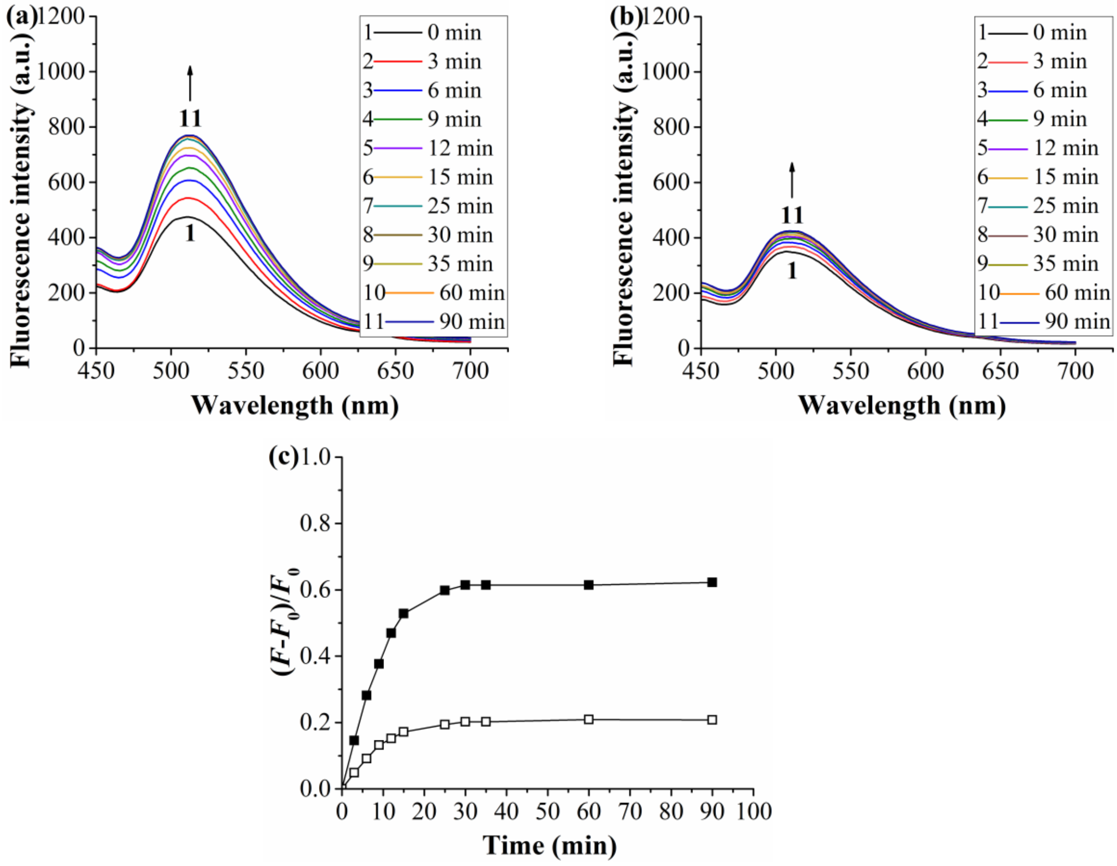

Figure S5. (a,b) Fluorescence spectra of the hydrophilic fluorescent solid HA-MIP (a)/CP (b) (0.25 mg/mL) after their incubation with a HA solution (20 μM) in the artificial urine at 25 °C for different times. (c) Optosensing kinetics of the hydrophilic fluorescent solid HA-MIP (filled symbol)/CP (open symbol) in a HA solution (20 μM) in the artificial urine at 25 °C [derived from Figure S5a,b; F_t and F_0 in $(F_t - F_0)/F_0$ are the fluorescence intensity of the NBD unit (at 514 nm) at a time of t and 0, respectively].

and S5c). An obvious increase in the fluorescence intensity at 514 nm was observed for both the hydrophilic fluorescent hollow and solid HA-MIPs/CPs after their binding HA in the artificial urine. Their fluorescence enhancement [i.e., $(F\text{-}F_0)/F_0$, where F and F_0 are the fluorescence intensities of the NBD unit (at 514 nm) in the presence and absence of HA, respectively] could reach maximum around 12 and 30 min, respectively (Figures 3c and S5c).

Spectrofluorimetric titration of the hydrophilic fluorescent solid and hollow HA-MIPs/CPs in the artificial urine

The spectrofluorimetric titration experiments of the hydrophilic fluorescent solid and hollow HA-MIPs/CPs in the artificial urine were carried out as follows: the studied HA-MIP or CP (0.25 mg/mL, the concentration of the hollow MIP/CP was calculated by using the weight of their solid counterparts before their etching) was first dispersed in 1 mL of a series of HA solutions in the artificial urine with different concentrations (C = 0, 5, 10, 15, 20, 30, 50, and 100 μM). After these mixtures were incubated at 25 ^{o}C for 2 h, their fluorescence spectra were measured (Figures 4a,b and S6a,b). The fluorescence intensities of NBD fluorophores around 514 nm were selected for the optosensing analyses. The linear calibration curves were obtained for the studied HA-MIPs/CPs by plotting the fluorescence enhancement values [i.e., $(F\text{-}F_0)/F_0$] versus HA concentrations (i.e., fitting the above spectrofluorimetric titration results with Stern-Volmer equation).

Optosensing selectivity of the hydrophilic fluorescent solid and hollow HA-MIPs/CPs toward HA in the artificial urine

The optosensing selectivity of the hydrophilic fluorescent solid and hollow HA-MIPs/CPs was studied as follows: the studied HA-MIP or CP (0.25 mg/mL, the concentration of the hollow MIP/CP was calculated by using the weight of their solid counterparts before their etching) was dispersed in 1 mL of a HA, 3-MHA, 4-AHA, or Tyr solution ($C_{\text{HA, 3-MHA, 4-AHA, or Tyr}}$ = 20 μM) or a mixed solution of HA (20 μM) with 40 μM of 3-MHA, 4-AHA, or Tyr in the artificial urine. After these mixtures were incubated at 25 ^{o}C for 2 h, their fluorescence spectra were measured, from which the fluorescence enhancement values [i.e., $(F\text{-}F_0)/F_0$] of the studied HA-MIPs and CPs were derived and used for analyses (Figures 5 and S7).

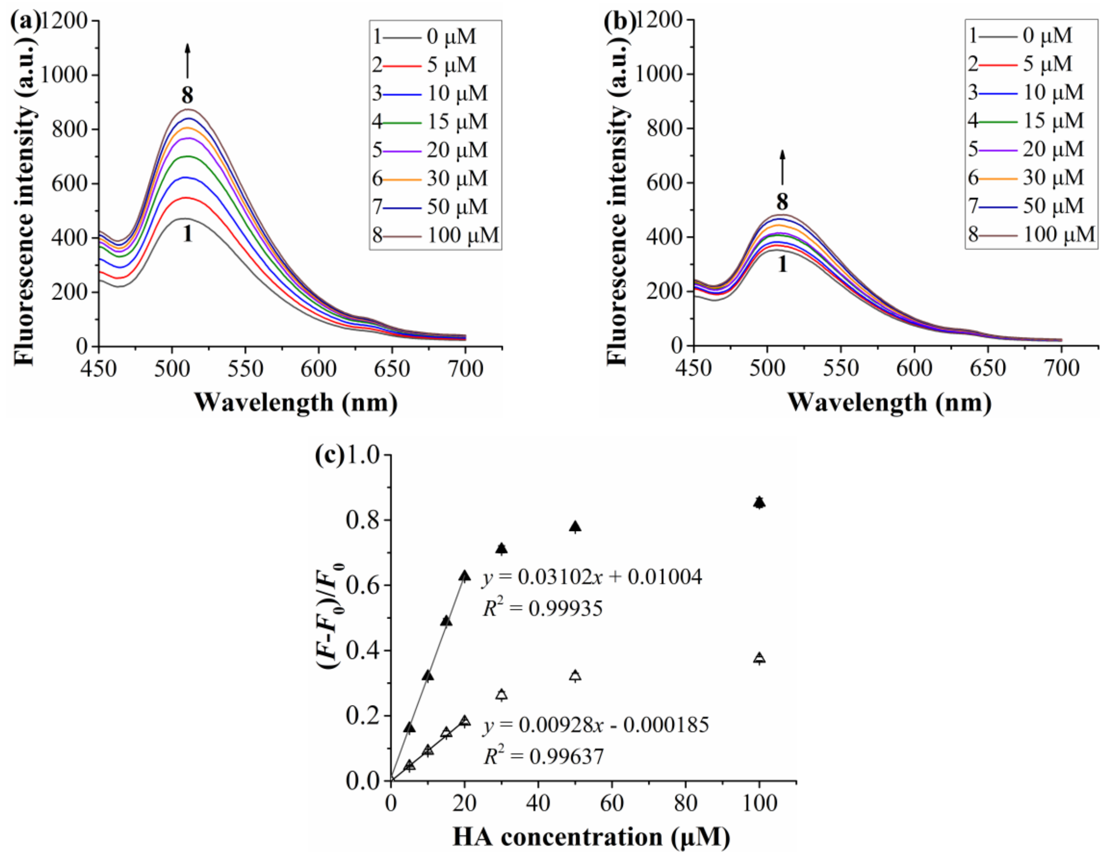

Figure S6. (a,b) Fluorescence spectra of the hydrophilic fluorescent solid HA-MIP (a)/CP (b) upon their exposure to different concentrations of HA in the artificial urine at 25 °C for 2 h (MIP/CP concentration: 0.25 mg/mL). (c) Dependence of the fluorescence enhancement [$(F\text{-}F_0)/F_0$] of the hydrophilic fluorescent solid HA-MIP (filled symbol)/CP (open symbol) on the HA concentration (derived from Figure S6a,b).

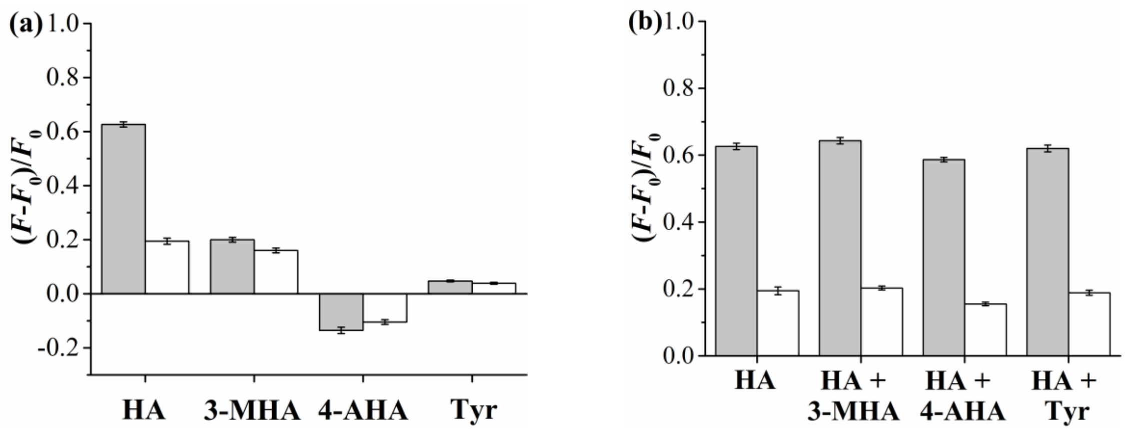

Figure S7. Fluorescence enhancement of the hydrophilic fluorescent solid HA-MIP (filled column)/CP (open column) upon exposure to a HA, 3-MHA, 4-AHA, or Tyr solution ($C_{\text{HA, 3-MHA, 4-AHA, or Tyr}}$ = 20 μM) (a) or to a HA solution (20 μM) in the presence of 40 μM of HA, 3-MHA, 4-AHA, or Tyr (b) in the artificial urine at 25 °C for 2 h (MIP/CP concentration: 0.25 mg/mL).

Photostability of the hydrophilic fluorescent solid and hollow HA-MIPs/CPs

The photostability of the hydrophilic fluorescent solid and hollow HA-MIPs/CPs was determined by measuring the fluorescence intensity changes of their dispersed mixtures in pure water (0.25 mg/mL, the concentration of the hollow HA-MIP/CP was calculated by using the weights of their solid counterparts before etching) over time at 25 °C under air atmosphere (Figure S8).

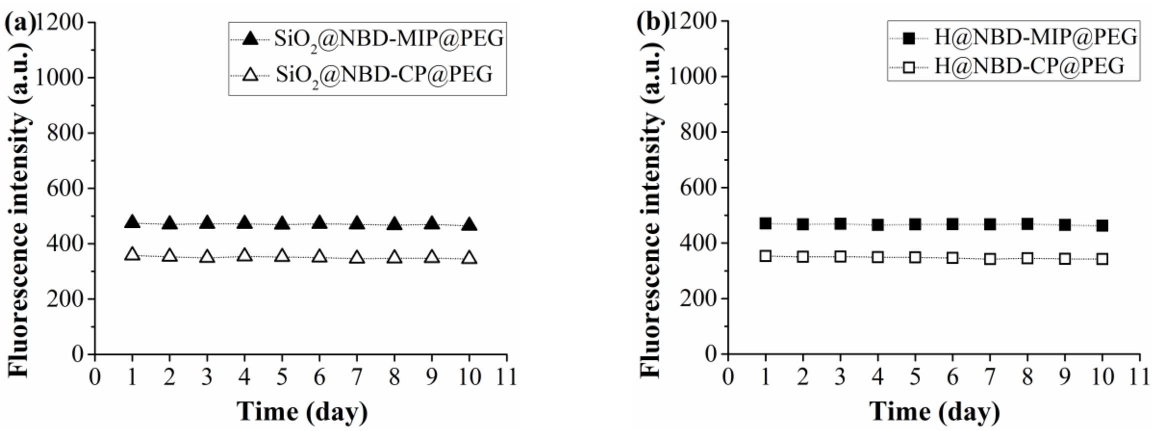

Figure S8. The fluorescence intensity changes (around 514 nm) of dispersed mixtures of SiO_2@NBD-MIP/CP@PEG (a) or H@NBD-MIP/CP@PEG (b) in pure water over time at 25 °C under air atmosphere (HA-MIP/CP concentration: 0.25 mg/mL, the concentration of the hollow HA-MIP/CP was calculated by using the weights of their solid counterparts before etching).

Reusability of the hydrophilic fluorescent solid and hollow HA-MIPs/CPs

The reusability of the hydrophilic fluorescent solid and hollow HA-MIPs/CPs was assessed by measuring the fluorescence intensity changes of their dispersed mixtures in the artificial urine after their consecutive HA adsorption-desorption cycles as follows: 0.25 mg of solid or hollow HA-MIP/CP (the mass of the hollow HA-MIP/CP was calculated by using the weights of their solid counterparts before etching) was first dispersed in 1 mL of the artificial urine, and the fluorescence intensity of the mixture was determined. After centrifugating the above mixture and removing the supernatant, the resulting MIP/CP was washed with pure water thrice and then with methanol twice, followed by drying with argon flowing. The dried MIP/CP was then incubated with a HA solution in the artificial urine (20 μM, 1 mL) at 25 °C for 1 h, and the fluorescence intensity of the mixture was determined. Afterwards, the above

mixture was centrifuged again, the resulting MIP/CP with bound HA was first washed with pure water thrice and then thoroughly with methanol and acetic acid (9:1 v/v) until no HA was detectable with HPLC in the washing solution. The resulting MIP/CP was then dried with argon flowing and the dried MIP/CP was dispersed in 1 mL of the artificial urine. The fluorescence intensity of the above mixture was again determined. The above procedure was repeated for 10 cycles and the results are presented in Figure S9a,b.

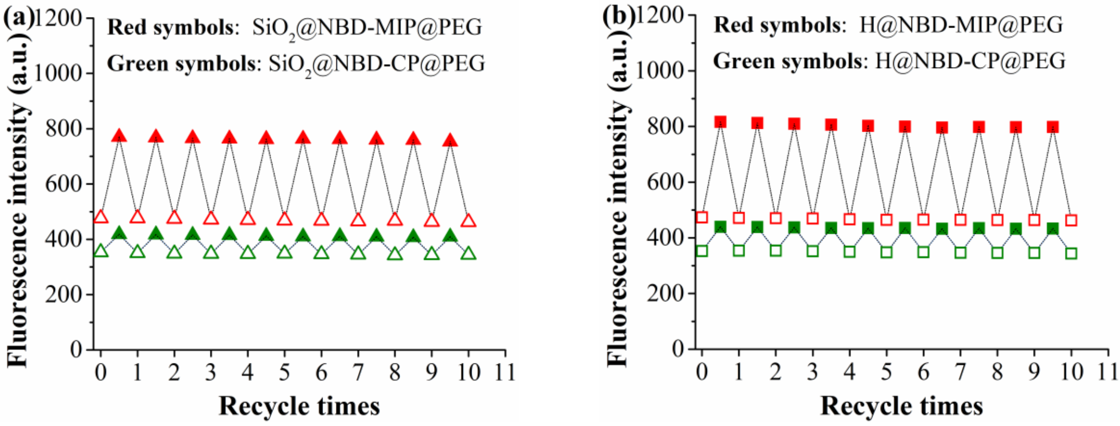

Figure S9. Fluorescence intensity changes (around 514 nm) of SiO_2@NBD-MIP/CP@PEG (a) or H@NBD-MIP/CP@PEG (b) upon desorption (empty) and adsorption (filled) of HA (20 μM) in the artificial urine during their 10 regeneration cycles (HA-MIP/CP concentration: 0.25 mg/mL, the concentration of the hollow HA-MIP/CP was calculated by using the weights of their solid counterparts before etching).

Optosensing assay of HA in the undiluted human urine with the hydrophilic fluorescent hollow HA-MIP

The optosensing assay of HA in the undiluted human urine with hydrophilic fluorescent hollow HA-MIP was carried out as follows:

1) The calibration curve for the optosensing of HA was first determined by performing the spectrofluorimetric titration experiments of the hydrophilic fluorescent hollow HA-MIP in the artificial urine as described in the section "***Spectrofluorimetric titration of the hydrophilic fluorescent solid and hollow HA-MIPs/CPs in the artificial urine***" (Figure 4c).

2) The optosensing assay of HA in the undiluted human urine was performed by first incubating a series of mixtures of the hydrophilic fluorescent hollow HA-MIP (0.25 mg/mL, the concentration of the hollow MIP was calculated by using the weight of its solid

counterpart before etching) in the undiluted human urine samples (1 mL) with different spiking levels of both HA and its mixtures with several analogues at 25 ^{o}C for 15 min and then measuring their fluorescent spectra. The amounts of HA inside the above-studied undiluted human urine samples were readily derived by fitting their fluorescence intensity data into the above-obtained linear calibration equation for the hydrophilic fluorescent hollow HA-MIP (Table 2).

Table S2. Performance comparison of our hydrophilic fluorescent hollow HA-MIP optosensor with other previously reported MIP-based detection systems for HA.

Analytical method [a]	Sample	Linear range	LOD	Recovery (%)	RSD (%)	Ref.
Solid-phase extraction (SPE)/HPLC-UV	Human urine (filtered through Whatman paper No. 42)	0.3-7500 μg/L (0.0017-41.86 μM)	0.15 μg/L (0.84 nM)	88.0-104.0	< 6.1	[11]
SPE/LC-MS/MS	Human urine (filtered through a 0.22 μm PTFE membrane)	0.5-10000 μg/L (0.0028-55.81 μM)	89 ng/L (0.50 nM)	91.4-109.1	6.4-9.6 (intra-day) 9.2-11.5 (inter-day)	[12]
SPE/micellar electrokinetic chromatography (MEKC)	Human urine (without pretreatment)	0.5-5.0 g/L (2.79-27.91 mM)	0.15 g/L (0.84 mM)	-	< 16	[13]
Micro-extraction by packed sorbent (MEPS)/LC-MS/MS	Plasma and urine (pretreated to remove proteins with acetonitrile)	1-1000 nM	0.3 nM	91-96	1.1-7.1	[14]
Hollow fiber based liquid-phase microextraction/ LC-MS/MS	Human plasma and urine [pretreated to remove proteins with 25 mM ammonium acetate (pH 5.0)]	1-2000 nM	0.3 nM	97-104	1.2-4.1	[15]
Electrochemical sensing	Human serum (pretreated to remove proteins with methanol) and diluted human urine	0.05-40 nM and 40-500 nM	0.012 nM	96.0-105.0	1.2-3.2	[16]
Direct fluorescent optosensing	Human urine (without any pretreatment)	0-20 μM	0.097 μM	96.0-102.0	0.6-4.0	This work

[a] SPE separation normally requires four steps (i.e., column condition, sample upload, wash, and elution), which is rather time-consuming and tedious. In addition, many SPE parameters (e.g., the sample pH, the amount of the sorbent, and the washing and elution solvent volumes) also need optimization prior to the sample extraction.

References

1. Zhang, H.; Klumperman, B.; Ming, W.; Fischer, H; van der Linde, R. Effect of Cu(II) on the kinetics of the homogeneous atom transfer radical polymerization of methyl methacrylate. *Macromolecules* **2001**, 34, 6169-6173.
2. Unsal, E.; Elmas, B.; Çağlayan, B.; Tuncel, M.; Patir, S.; Tuncel, A. Preparation of an ion-exchange chromatographic support by a “grafting from” strategy based on atom transfer radical polymerization. *Anal. Chem.* **2006**, 78, 5868-5875.
3. Ciampolini, M.; Nardi, N. Five-coordinated high-spin complexes of bivalent cobalt, nickel, and copper with tris(2-dimethylaminoethyl)amine. *Inorg. Chem.* **1966**, 5, 41-44.
4. Wan, W.; Biyilal, M.; Wagner, R.; Sellergren, B.; Rurack, K. Fluorescent sensory microparticles that “light-up” consisting of a silica core and a molecularly imprinted polymer (MIP) shell. *Angew. Chem. Int. Ed.* **2013**, 52, 7023-7027.
5. Li, Q.; Zhang, W.; Liu, X.; Zhang, H. Preparation of complex biological sample-compatible “turn-on”-type ratiometric fluorescent molecularly imprinted polymer microspheres via one-pot surface-initiated ATRP. *Microchim. Acta* **2022**, 189, 464.
6. Chutipongtanate, S.; Thongboonkerd, V. Systematic comparisons of artificial urine formulas for in vitro cellular study. *Anal. Biochem.* **2010**, 402, 110-112.
7. Li, C.; Ma, Y.; Niu, H.; Zhang, H. Hydrophilic hollow molecularly imprinted polymer microparticles with photo- and thermoresponsive template binding and release properties in aqueous media. *ACS Appl. Mater. Interfaces* **2015**, 7, 27340-27350.
8. Zheng, C.; Zhou, Y.; Jiao, Y.; Zhang, H. Narrow or monodisperse, physically cross-linked, and “living” spherical polymer particles by one-stage RAFT precipitation polymerization. *Macromolecules* **2019**, 52, 143-156.
9. Boonpangrak, S.; Whitcombe, M.J.; Prachayasittikul, V.; Mosbach, K.; Ye, L. Preparation of molecularly imprinted polymers using nitroxide-mediated living radical polymerization. *Biosens. Bioelectron.* **2006**, 22, 349-354.
10. Hishiya, T.; Shibata, M.; Kakazu, M.; Asanuma, H.; Komiyama, M. Molecularly imprinted cyclodextrins as selective receptors for steroids. *Macromolecules* **1999**, 32, 2265-2269.

11. Arabi, M.; Ghaedi, M.; Ostovan, A. Water compatible molecularly imprinted nanoparticles as a restricted access material for extraction of hippuric acid, a biological indicator of toluene exposure, from human urine. *Microchim. Acta* **2017**, 184, 879-887.
12. Hu, C.; Yang, Z.; Yan, F.; Sun, B. Extraction of the toluene exposure biomarkers hippuric acid and methylhippuric acid using a magnetic molecularly imprinted polymer and their quantitation by LC-MS/MS. *Microchim. Acta* **2019**, 186, 135.
13. Boscari1, C.N.; Mazzuia, G.R.; Wisniewski, C.; Borges, K.B.; Figueiredo, E.C. Molecularly imprinted probe for solid-phase extraction of hippuric and 4-methylhippuric acids directly from human urine samples followed by MEKC analysis. *Electrophoresis* **2017**, 38, 1083-1090.
14. Moein, M.M.; El-Beqqali, A.; Javanbakht, M.; Karimi, M.; Akbari-adergani, B.; Abdel-Rehim, M. On-line detection of hippuric acid by microextraction with a molecularly-imprinted polysulfone membrane sorbent and liquid chromatography-tandem mass spectrometry. *J. Chromatogr. A* **2014**, 1372, 55-62.
15. Moein, M.M.; Javanbakht, M.; Karimi, M.; Akbari-adergani, B.; Abdel-Rehim, M. Three-phase molecularly imprinted sol-gel based hollow fiber liquid-phase microextraction combined with liquid chromatography-tandem mass spectrometry for enrichment and selective determination of a tentative lung cancer biomarker. *J. Chromatogr. B* **2015**, 995-996, 38-45.
16. Karazan, Z.M.; Roushani, M. A new method for electrochemical determination of Hippuric acid based on molecularly imprinted copolymer. *Talanta* **2022**, 246, 123491.
